# Double-encapsulated red-emitting formamidinium lead halide perovskite nanocrystals for fluorescence sensing and lighting applications†

**DOI:** 10.1039/d5na00412h

**Published:** 2025-07-14

**Authors:** Kajol Sahoo, Latika Juneja, Ramakanta Naik, Saikat Bhaumik

**Affiliations:** a Department of Engineering and Materials Physics, Institute of Chemical Technology IndianOil Odisha Campus Bhubaneswar Odisha 751013 India; b Department of Physics, Indian Institute of Technology Guwahati Assam 781039 India s.bhaumik@iitg.ac.in

## Abstract

In recent years, metal halide perovskite nanocrystals (NCs) have offered several optoelectronic and sensing applications due to their unique photophysical properties. Formamidinium (FA) based perovskite NCs exhibit better thermal and structural stability than the corresponding volatile methylammonium (MA)-based counterpart, which quickly decomposes to release gaseous methylamine. FA-based perovskite NCs also demonstrate fantastic responses towards different environmental stimuli, which is helpful for different sensing applications. However, FAPbI_3_ NCs suffer from phase instability and degrade very fast under external stimuli. Here, we synthesized mixed halide FAPb(Br/I)_3_ NCs by partially replacing I-ions with Br-ions to address the key challenge of phase instability of FAPbI_3_ NCs. Furthermore, with a surface modification approach such as encapsulating the surface of NCs with silica and also a double-encapsulated silica-polymethyl methacrylate (PMMA) polymer, we enhanced the stability of the NCs against heat, ion migration, and UV irradiation. These double-coated red-emitting FAPb(Br/I)_3_ NCs (emission peak ∼ 642 nm) were tested for temperature sensing, exhibiting a relative sensitivity (*S*_r_) of ∼12.5% K^−1^. We also prepared fluorescent humidity sensors that revealed the lowest detection limit of ∼5% relative humidity (RH). Finally, down-converted WLEDs were fabricated using double-coated green-emitting Cs-doped FAPbBr_3_ NCs and red-emitting FAPb(B/I)_3_ NCs in thin-film form and embedded on a blue LED chip. These results will boost the development of high-performance sensors and lighting technologies.

## Abstract 2

Red-emitting FAPb(Br/I)_3_ nanocrystals were double-encapsulated with silica and a PMMA to improve the stability, and demonstrated for optical sensing and lighting applications.

## Graphical abstract

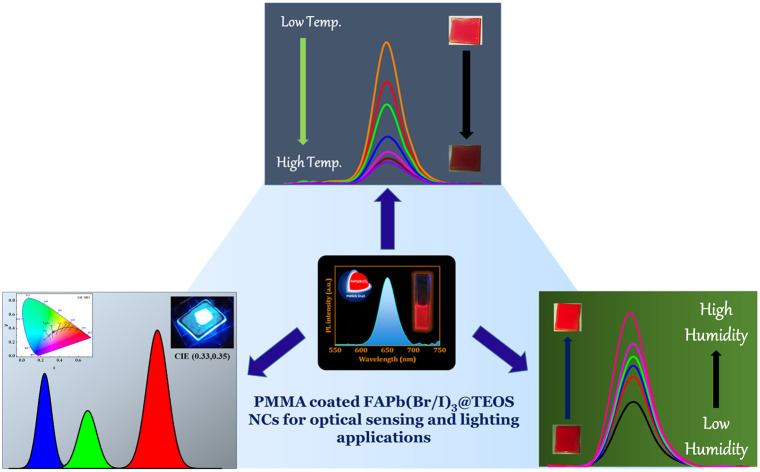

## 1. Introduction

The modern world is grappling with numerous challenges related to pollution, climate change, the rise in global temperature, food safety, and many more. In this direction, the emergence of innovative sensing techniques, such as electrochemical and optical sensors, holds significant promise in detecting the changes in material properties when exposed to specific chemical or physical stimuli, thereby tackling many of the challenges we face today.^1–3^ These advanced sensors transform fields such as environmental monitoring, healthcare, food safety, and industrial processes by providing real-time, accurate data on various chemical and environmental parameters. Various sensing techniques, such as optical, optoelectronic, capacitive, resistive, quartz crystal microbalance (QCM), and photonics sensors, have been developed for effective signal monitoring. Each technology offers unique advantages and is suited to specific use depending on factors such as sensitivity, size, cost, environmental considerations, *etc.* Fluorescence sensing is considered a more versatile, non-invasive, and cost-effective method.

Recently, organic–inorganic lead halide perovskite [APbX_3_: X= Cl, Br, I] nanocrystals (NCs) have emerged as promising materials for various optoelectronic devices, lasing, bio-imaging, fluorescence-based sensing, *etc.*^4–10^ The unique properties of these NCs, such as high photoluminescence quantum yield (PLQY), narrow emission width, tunable bandgap, multiphoton absorption capability, and high signal-to-noise ratio, make them well-suited for various applications.^4–10^ Development of perovskite light-emitting diodes (PeLEDs) is a groundbreaking advancement in display technology, especially for color conversion.^11,12^ The superior performance of PeLEDs over organic LEDs and quantum dot LEDs is owing to some of their remarkable properties, particularly in terms of the wider color gamut, high color purity, narrow full-width half maxima (FWHM), defect-tolerant nature, low turn-on voltage, *etc.* Usually, the emission peak position of methylammonium lead bromide (MAPbBr_3_) and cesium lead bromide (CsPbBr_3_) NCs is located below 520 nm wavelength, which is smaller than that of the required National Television System Committee (NTSC) standard green emission.^13,14^ However, formamidinium lead halide (FAPbX_3_) NCs emit ultrapure spectral emissions, making them promising in display and lighting technologies.^15–17^

MAPbX_3_ perovskite materials have been extensively explored for different optoelectronic applications. However, MAPbX_3_ materials possess undesired intrinsic thermal instability because of the volatile MA^+^-ions, which decompose to release gaseous methylamine.^18^ Still, the NCs face significant stability issues. Their ionic nature makes them highly susceptible to phase transitions or chemical oxidation when exposed to external stresses such as heat, moisture, halide migration, and UV irradiation, severely limiting their practical applications.^10,19,20^ FA cation-based perovskites can be an effective material to replace MAPbX_3_. FAPbX_3_ perovskite materials exhibit better light, air, and thermal stabilities, making them suitable for various applications.^21–24^ Ongoing research focuses on improving their long-term stability to make perovskite NCs more viable for real-world commercial needs. Researchers have developed several strategies, which include doping, surface engineering, embedding perovskites within protective matrices, *etc.* Doping with different ions at A or Pb-sites is a common strategy to improve the phase stability of perovskite NCs.^25–28^ Another important strategy to improve structural stability is coating the perovskite core with different higher bandgap materials. Silica, polymers, metal–organic frameworks (MOFs), and metal chalcogenides are some of the most used encapsulating materials, which protect the perovskite core from destabilization or degradation.^29–31^ Among them, the double encapsulation technique helps significantly improve the stability and PL intensity of NCs.^32–35^

Many research articles have been published showing the sensing performance of metal halide perovskite NCs. These materials have been extensively used to detect heavy metal ions and in gas, temperature, humidity, and radiation sensing applications.^36–40^ Nowadays, optical sensors for thermometry have become a key area of interest in many fields due to their numerous advantages. These sensors offer several benefits that make them particularly attractive for various applications, including scientific research, industrial processes, medical diagnostics, and environmental monitoring, because of their remarkable properties, such as high sensitivity, cost-effectiveness, and less invasive properties. Besides temperature, relative humidity (RH) also plays a key role in maintaining optimal conditions for processes, products, and the environment. So, accurate monitoring is essential for both quality control and operational efficiency. Perovskite NCs, being ionic in nature, exhibit a more significant response to humidity because humidity exposure can change the crystal structure and conductivity of the material, leading to a measurable change in their optical, electrical, and dielectric properties.^41^ Xing *et al.* reported that the nanoarray of Cs_4_PbBr_6_ was being used as an optical humidity sensor with a detection limit of 9.5%.^42^ Yella *et al.* reported a self-powered ion voltaic response of 0-D MA_4_PbI_6_ NCs to relative humidity ranging from 10–90%.^43^ There is limited literature on FA-based perovskite NCs published as optical temperature and humidity sensors. A red-emitting light source is recommended because less scattering of light results in deeper penetration in different living cells. Noteworthy FA-based red-emitting perovskite NC development is important to understanding their photophysical properties and corresponding applications in various research fields.

We report synthesizing red-emitting FAPb(Br/I)_3_ NCs using the room-temperature ligand-assisted reprecipitation (LARP) synthesis method. To stabilize the black phase of FAPbI_3_ NCs, we partially replace I-ions with smaller Br-ions inside the perovskite crystal. We encapsulated them with silica and a methyl methacrylate (PMMA) polymer to further improve their stability and luminescence intensity. We demonstrated the capability of these double-coated NC films for optical temperature and humidity sensing. We also synthesized green-emitting double-coated Cs-doped FAPbBr_3_ NCs. We fabricated color-converting WLEDs by incorporating green and red-emitting FA-based double-coated NC films placed on a blue LED chip operated through an external bias.

## 2. Experimental section

### 2.1. Materials

Cesium bromide (CsBr, 99.999% trace metals basis), lead bromide (PbBr_2_, 99.999% trace metals basis), lead iodide (PbI_2_, 99%), PMMA (MW-350 000, 99.9%), oleic acid (OA, 90%), oleylamine (OAm, 70%), *N*,*N*′-dimethylformamide (DMF, anhydrous, 99.8%), tetraethylorthosilicate (TEOS, reagent grade, 98%), chloroform (CHCl_3_, 99.9%), toluene (anhydrous, 99.8%), acetonitrile (ACN, 99.95%), and tetrabutylammonium chloride (TBA-Cl, ≥97%) were purchased from Sigma-Aldrich company. Formamidinium bromide (FABr, >99.99%) and formamidinium iodide (FAI, >99.99%) were purchased from Greatcell Solar Material Company. All the chemicals were used without further purification.

### 2.2. Synthesis of OA and OAm capped FAPb(Br/I)_3_ NCs

We synthesized OA and OAm capped FAPb(Br/I)_3_ NCs by following the reported LARP synthesis method.^44^ Here, 0.05 mmol FAI, 0.0375 mmol PbBr_2_, 0.0125 mmol PbI_2_, 100 μL OA, 20 μL OAm, and 500 μL DMF were dissolved together in a glass vial to form the final precursor solution. Then 250 μL of the final precursor was injected dropwise into a round-bottom flask containing 5 mL CHCl_3_, which was kept under vigorous stirring at room temperature (RT). The reaction continued for 5 min for the nucleation and growth of the NCs. Later, toluene and ACN were added in a 1 : 1 ratio to the NCs, and the mixture was centrifuged at 9000 rpm for 2 min in a centrifuge tube. Finally, the precipitate was dispersed in toluene and named FP(B/I)@O NCs.

### 2.3. Synthesis of silica-coated FAPb(Br/I)_3_ NCs

The same method was adopted to prepare the final precursor solution for silica-coated FAPb(Br/I)_3_ NC synthesis.^36^ Here, 250 μL of the final precursor was added to the round-bottom flask containing 5 mL CHCl_3_ with the desired amount of TEOS solvent. The reaction continued for 4 hours under vigorous stirring conditions at RT. We optimized the silica encapsulation around the NCs by varying the amount of TEOS to 50, 100, and 150 μL. The NC mixture was purified in the same way as discussed earlier. The silica-coated FAPb(Br/I)_3_ NCs synthesized using 100 μL TEOS were assigned as FP(B/I)@S NCs.

### 2.4. Encapsulation of PMMA over silica-coated FAPb(Br/I)_3_ NCs

50 mg PMMA powder was mixed in 1 mL toluene upon heating at 80 °C.^36^ Then, 2 mL of silica-coated NCs was added into the PMMA solution and mixed by stirring for 2 hours at RT. The precipitate of the NCs was collected by centrifugation at 9000 rpm for 2 min and dispersed in toluene. The polymer-coated NCs were renamed FP(B/I)@S@P NCs.

## 3. Results and discussion

**Fig. 1:**
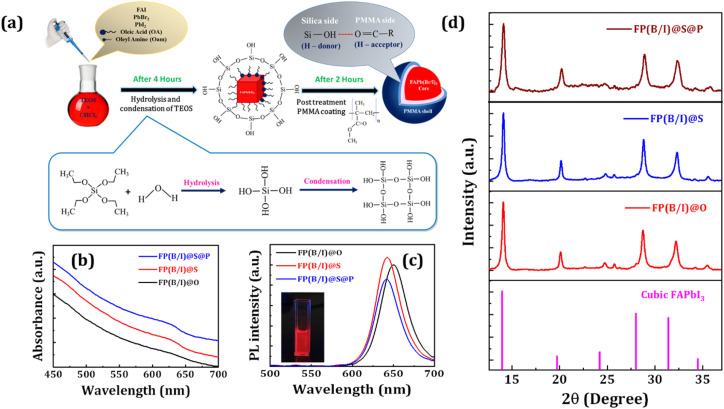
(a) Schematic diagram representing the LARP synthesis method of perovskite NCs and sequential encapsulation of TEOS and PMMA around FAPb(Br/I)_3_ NCs. (b) UV-Vis absorption spectra, (c) PL spectra, and (d) XRD patterns of different FAPb(Br/I)_3_ NCs, as represented in legends. The inset of figure (c) shows a photographic image of the FP(B/I)@S@P NC dispersion under a UV lamp. The bottom of (d) presents the standard XRD pattern of the cubic FAPbI_3_ phase.

We synthesized FAPb(Br/I)_3_ NCs with silica coating while varying the TEOS amount from 50 to 150 μL. The corresponding absorption and PL spectra of these NCs are presented in Fig. S1a and b (see the ESI†). All silica-coated NCs emit red color emission, among which FP(B/I)@S NCs exhibit the highest PL intensity. Here, FP(B/I)@S NCs were synthesized with optimal 100 μL TEOS that passivates the surface of the NCs and improves radiative recombination. However, with a higher amount of silica content, the PL intensity decreased because of steric hindrance from excessive Si–O–Si bonding. A schematic diagram depicting the synthesis of FP(B/I)@S@P NCs is shown in Fig. 1a. The UV-Vis spectrum of OA and OAm capped FAPb(Br/I)_3_ (*i.e.*, FP(B/I)@O) NCs is presented in Fig. 1b and exhibits an absorption edge close to 658 nm. With the silica coating, the absorption edge of the NCs shifted towards the lower wavelength region. The FP(B/I)@S NCs exhibit an absorption edge at around 648 nm. However, the polymer coating around the FP(B/I)@S (*i.e.*, FP(B/I)@S@P) NCs does not shift the absorption edge significantly, which shows an absorption edge of ∼647 nm. The optical bandgaps of the NCs were obtained from the Tauc plots, and the corresponding spectra are shown in Fig. S2 in the ESI.† For FP(B/I)@O NCs, the bandgap was calculated to be around 1.83 eV. The optical bandgaps of FP(B/I)@S and FP(B/I)@S@P NCs slightly increased because of the quantum confinement effect. The bandgap of double-coated FP(B/I)@S@P NCs is estimated to be ∼1.86 eV.

The emission spectra of these NCs reflect similar features (see Fig. 1c). The PL spectrum of FP(B/I)@O NCs reveals an emission peak at 650 nm (FWHM ∼ 39 nm) with a PLQY of ∼20%. The PL peak position for FP(B/I)@S NCs is 642 nm (FWHM ∼ 36 nm) and exhibits a PLQY of ∼21%. The PL spectrum remains almost unaffected after polymer coating around the silica-coated NCs. However, PL intensity decreased moderately, and the PLQY was calculated to be ∼16%. We also carried out time-resolved photoluminescence (TRPL) measurements to probe the photocarrier recombination dynamics before and after double-coating, and the corresponding graphs for FP(B/I)@O and FP(B/I)@S@P NCs are provided in Fig. S3 in the ESI.† The average lifetime of the NCs was calculated by using the bi-exponential decay function, using the formula:
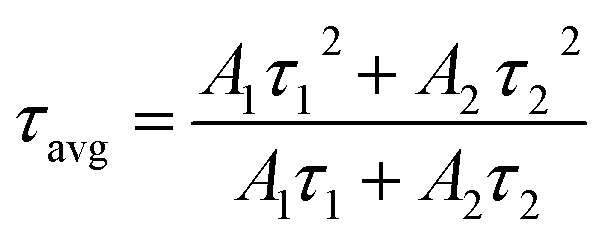


The calculated lifetime of both samples is given in Table S1 in the ESI.† The average lifetime of the FP(B/I)@O NCs was found to be 46.96 ns. After double-coating, the average lifetime of the FP(B/I)@S@P sample reduced to 40.48 ns. The decrease in the lifetime in the double-coated sample is consistent with the PL emission intensity of the corresponding NCs. This contributes to the formation of a non-radiative decay channel created by more trap states due to double-coating.

X-ray diffraction (XRD) patterns of all NCs are shown in Fig. 1d, which depicts almost similar spectra. The prominent XRD peaks at 14.09°, 20.10°, 24.7°, 28.73°, and 32.23°, corresponding to diffractions from the (100), (110), (111), (200), and (210) lattice planes, signify the α-phase of the cubic FAPbI_3_ structure (*Pm*3̄*m* space group).^24^ However, these XRD peaks move to slightly higher angles from the standard diffraction pattern of the FAPbI_3_ phase because of the contraction of lattice spacing with partial substitution of Br-ions in place of I-ions.^32^

**Fig. 2:**
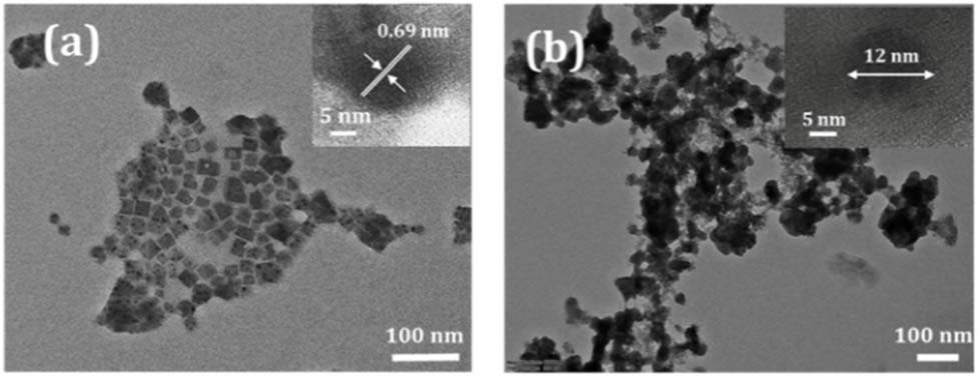
TEM images of (a) FP(B/I)@O and (b) FP(B/I)@S NCs. Insets of the figures present the HRTEM images of respective NCs.

**Fig. 3:**
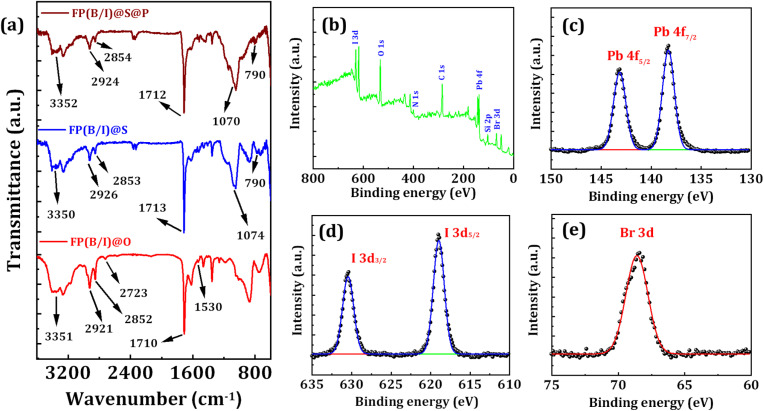
(a) FTIR spectra of different FAPb(Br/I)_3_ NCs as shown in the legend. (b) Core XPS spectrum and HR-XPS spectra of (c) Pb 4f, (d) I 3d, and (e) Br 3d chemical states of FP(B/I)@S@P NCs.

The morphological analysis of the NCs was performed through transmission electron microscope (TEM) and high-resolution transmission electron microscope (HRTEM) imaging. Fig. 2a presents the TEM and HRTEM (inset) images of FP(B/I)@O NCs, which are almost cubical in shape. The edge sizes of the NCs are distributed in the range of 5–35 nm, having an average edge size of ∼17 nm (see Fig. S4a in the ESI†). The interplanar spacing of the NCs was measured to be ∼0.69 nm, as shown in the inset of Fig. 2a, corresponding to the (100) lattice plane of the cubic FAPbI_3_ phase. The TEM image of FP(B/I)@S NCs (see Fig. 2b) reveals irregular particle shape. The blurred surface around the NCs originated from the compact silica shelling around the NCs.^32,33^ The particle size of the silica-coated NCs was estimated to be ∼12 nm, as observed from the HRTEM image (inset of Fig. 2b). The TEM image of double-coated FP(B/I)@S@P NCs exhibits a similar morphology to FP(B/I)@S NCs, signifying minimal influence on the particle shape and size after polymer shelling (see Fig. S4b in the ESI†). To identify the surface coating around the NCs, we performed FTIR analysis of all the NC films, and corresponding spectra are presented in Fig. 3a. The sharp peaks at around 1710 and 1530 cm^−1^ in FP(B/I)@O NC film spectra appear from the C=O and –NH functional groups of OA and OAm capping ligands, respectively.^32,33^ The additional peaks at 2723, 2852, and 2921 cm^−1^ correspond to the C–H stretching vibrations of OA and OAm ligands.^32,33^ The broad peak at around 3300–3400 cm^−1^ is associated with the N–H stretching vibration of OAm molecules. The FTIR spectra of FP(B/I)@S and FP(B/I)@S@P NC films seem almost similar for silica encapsulation. The two peaks at 790 and 1074 cm^−1^ appear from symmetrical stretching vibrations of Si–O bonds and anti-symmetrical stretching vibrations of the Si–O–Si groups of silica.^32,33^ Some additional peaks are visible from OA, OAm, and PMMA ligands.

We also performed X-ray photoelectron spectroscopy (XPS) of FP(B/I)@S@P NCs to get an insight into the surface elements in the NCs as presented in Fig. 3b. The HR-XPS spectra are well fitted and de-convoluted with the experimental value using a Gaussian fit. The HR-XPS spectrum of the Pb 4f orbital consists of two peaks at 138.2 eV and 143.1 eV originating from the spin orbit interaction of Pb 4f_7/2_ and 4f_5/2_ chemical states, as shown in Fig. 3c. Similarly, the I 3d orbital consists of two distinctive peaks at 618.9 and 630.4 eV corresponding to I 3d_5/2_ and 3d_3/2_ chemical states (see Fig. 3d). Fig. 3e presents the binding energy peak of the Br 3d orbital, which is located at 68.6 eV.^32,33^ The HR-XPS peaks for C, N, O, and Si chemical states are given in Fig. S5a–d in the ESI.† The de-convoluted XPS spectrum for the C 1s chemical state contains a couple of peaks at around 285 eV originating from C–C/C=N and C–N interaction of OA and FA^+^-molecules. The binding energy peak at 400.2 eV is associated with the N 1s chemical state. The peaks at 103.2 and 532.5 eV are associated with the Si 2p and O 1s chemical states of the silica shell present around the FP(B/I)@S@P NC core.^35^ The fitted parameters of each spectrum are summarized in Table S2 in the ESI.† Field emission scanning electron microscopy coupled with energy dispersive X-ray spectroscopy (FESEM-EDS) was performed to verify the elemental composition in the NC film. Fig. S6a–h† display the FESEM-EDS elemental mapping images of FP(B/I)@O NCs, which confirm the uniform distribution of Pb, I, Br, N, and O within the sample. The corresponding quantitative elemental analysis, in terms of both weight and atomic percentage, is provided in the inset of table Fig. S6i.† Similarly, the elemental mapping and quantitative data for silica-coated FP(B/I)@S and double-coated FP(B/I)@S@P NC films are shown in Fig. S7 and S8,† respectively. The presence of Si, in addition to C, O, N, Br, Pb, and I, is clearly observed in the coated samples, confirming successful silica encapsulation.

**Fig. 4:**
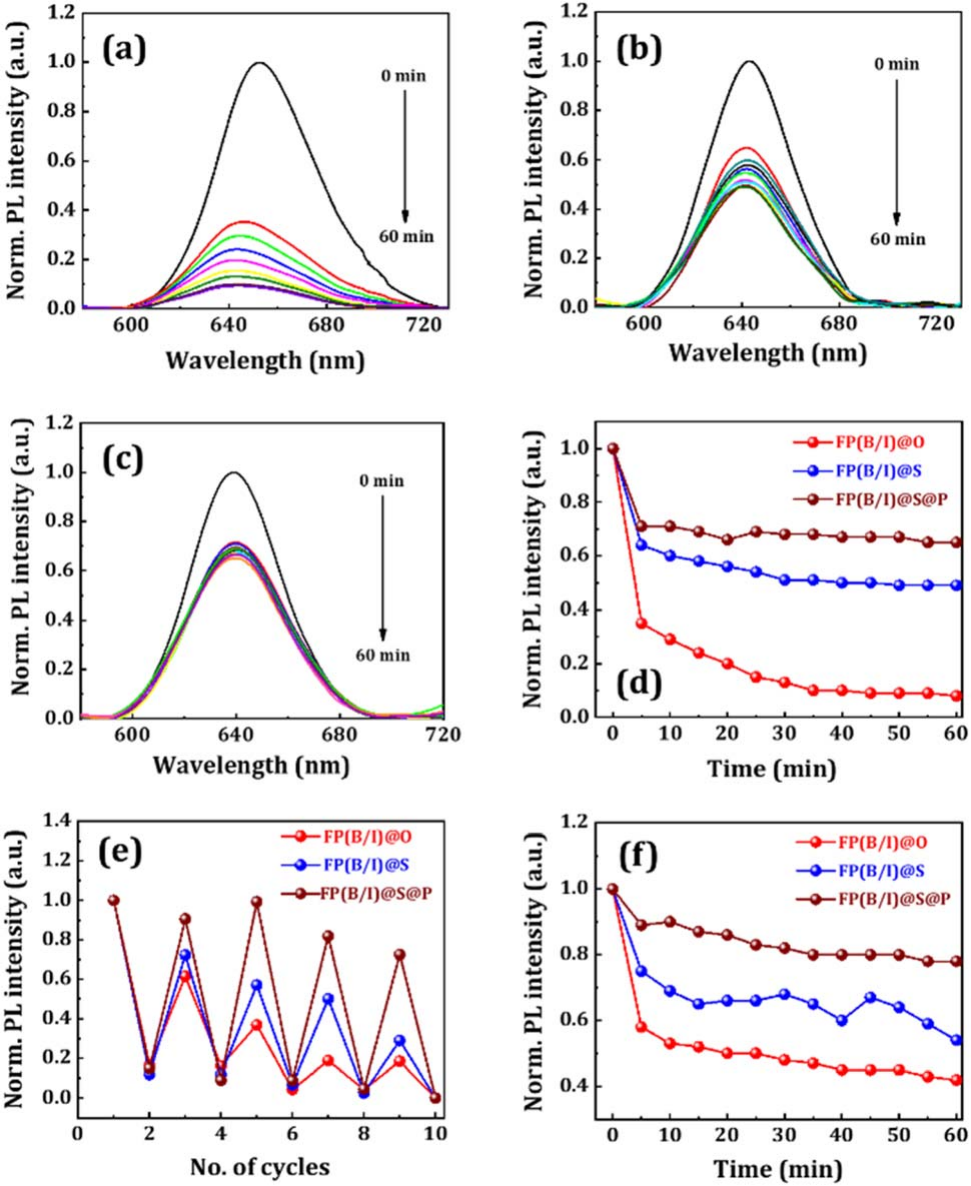
Change in PL intensity of (a) FP(B/I)@O, (b) FP(B/I)@S, and (c) FP(B/I)@S@P NC films kept at 70 °C. (d) The PL decay diagram depicts PL intensity variation over time, as represented in the legend. (e) Heating–cooling test of the NC films from room temperature to 70 °C. (f) UV stability test of the NC films when exposed to continuous UV irradiation for 1 hour.

The superior heat stability of NCs is desirable for different device-related applications. So, we executed the heat stability test of NC films by placing them on a hot plate and maintaining the temperature at 70 °C. Fig. 4a–c illustrate the film's emission spectral change, and Fig. 4d presents the corresponding decay diagrams during the heat stability test experiment. While heating the films, the emission intensity declines over time. The lattices of the perovskite materials vibrate more intensely at high temperatures, which leads to the formation of defects or strain within the crystal, which, in turn, act as non-radiative recombination centers and lead to quenching in emission intensity.^10,19,20^ The retention of PL intensity of FP(B/I)@O and FP(B/I)@S NC films was ∼8% and ∼49% of their initial values, respectively, after 60 min of heating. This indicates that the thermal stability of NCs has been improved by silica coating around the NCs, as the amorphous silica has low thermal conductivity due to its disordered structure and high surface area, which effectively scatters phonons, thereby providing better thermal stability.^45^ For the double-coated FP(B/I)@S@P NC sample, the retention of PL intensity was ∼65% after heating for the same period. Here, the thermoplastic polymer layer further protects the core from degradation due to lattice vibration when exposed to elevated temperatures for a considerable time.

We examined the PL stability of the films when exposed to multiple heating and cooling cycles, and the corresponding emission spectra are shown in Fig. 4e. By increasing the temperature from room temperature to 70 °C, the PL intensity of the FP(B/I)@O NC film reduced by ∼84%. The PL intensity gradually recovered when the films were removed from the hotplate and cooled to room temperature. The retention of PL intensity for the FP(B/I)@O NC film was ∼61% after the end of the 1^st^ cycle. We continued the experiment for five such cycles, and the retention of PL intensity for the film was ∼18% at the end of the 5^th^ cycle. The retention of PL intensity for the FP(B/I)@S and FP(B/I)@S@P NC films was ∼72% and 90% at the end of the 1^st^ heating–cooling cycle and ∼28% and ∼72% at the end of the 5^th^ cycle, respectively. The double-coating strategy enhances the overall stability of the perovskite NCs by insulating them from thermal, chemical, and physical degradation. UV light stability of all perovskite films was verified by exposing all films to 365 nm UV radiation for an hour. The spectral distribution with UV irradiation is shown in Fig. S9a–c in the ESI.† The corresponding decay diagrams are shown in Fig. 4f. For the FP(B/I)@O NC film, the PL intensity decreased by ∼58%, with the emission spectrum shifting towards a higher wavelength region upon continuous UV irradiation (see Fig. S9a, ESI†) because photo-induced halide segregation is more in mixed halide perovskite materials.^46^ For silica-coated FP(B/I)@S and double-coated FP(B/I)@S@P NC films, the decay in PL intensity was ∼46% and ∼22%, respectively, signifying that the NC coating enhances the photostability of the perovskite material. We also checked the ambient stability of the FP(B/I)@S@P NC film by monitoring the fluorescence behaviour under ambient conditions over an extended period. For this, we placed the NC film under ambient conditions (RH ∼ 50–60%), undisturbed. The corresponding photographic image of the NC film under a UV lamp with different time intervals is shown in Fig. S10 in the ESI.†

**Fig. 5:**
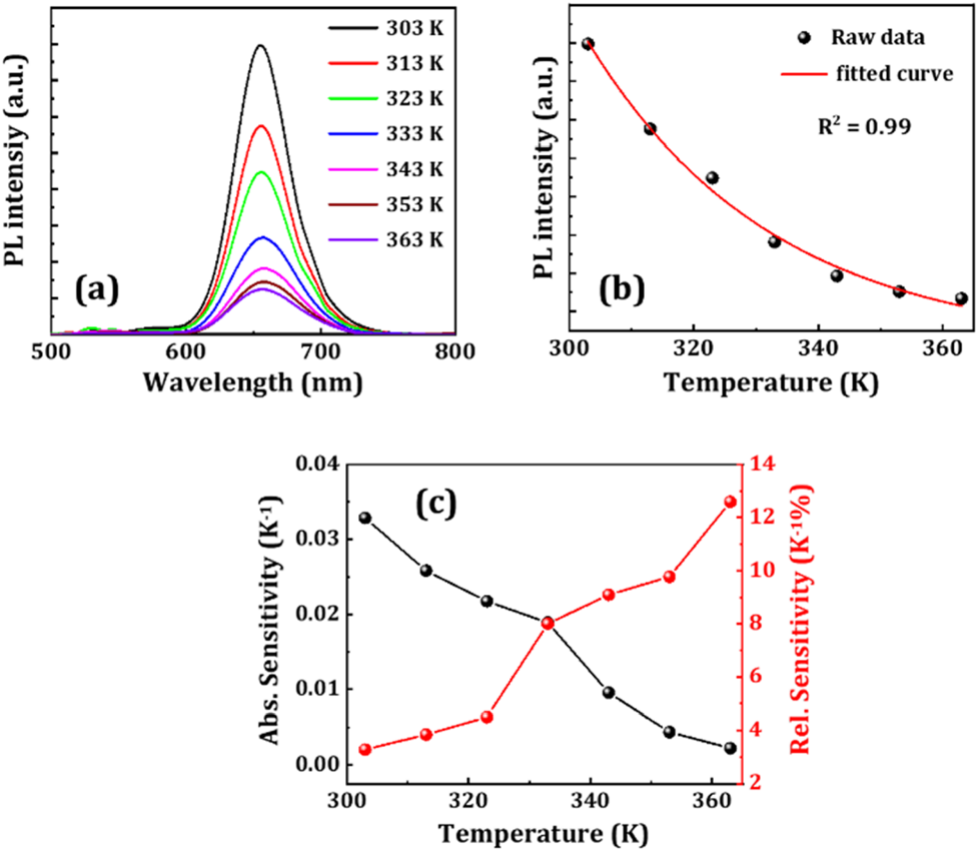
(a) Variation of PL intensity and (b) corresponding fitted data of the FP(B/I)@S@P NC film, while kept on a hot plate and heated at different temperatures as shown in legends. (c) Sensitivity plots of the temperature sensor.

Now, optical sensors for thermometry have gained significant attention because of their remarkable property of rapid response to the slightest variation in temperature. So, we explored the potential of FP(B/I)@S@P NCs as an optical sensor for detecting temperature variations. We recorded the change in fluorescence spectra of the FP(B/I)@S@P NC film with an increase in temperature starting from room temperature, *i.e.*, from 303 to 363 K. The corresponding change in spectral response is shown in Fig. 5a. It has been found that the fluorescence intensity started to quench with the increase in temperature. To provide more mechanistic insight about PL quenching of the FP(B/I)@S@P film with temperature, we verified the temperature-dependent broadening of spectra as shown in Fig. S11 (in the ESI†). The broadening of PL spectra is caused by the strong exciton–phonon coupling effect. A minor shift in the position towards the lower wavelength region, as evident from Fig. 5a, is attributed to the thermal expansion of the lattice altering the bond length and angle within the crystal lattice that arises because of the anharmonicity of the interatomic potential.^47^ The variation of PL intensity with temperature was fitted exponentially with a regression coefficient of 0.99 (see Fig. 5b). The sensitivity of the temperature sensor is defined in terms of absolute sensitivity (*S*_a_) and relative sensitivity (*S*_r_), which are the absolute values of the temperature derivative of the observable. The sensitivities are calculated from the following formulae:^48^
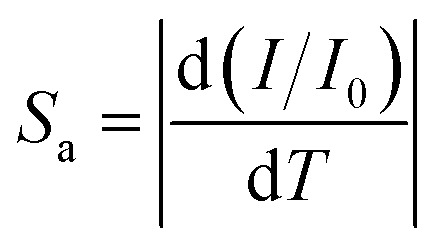

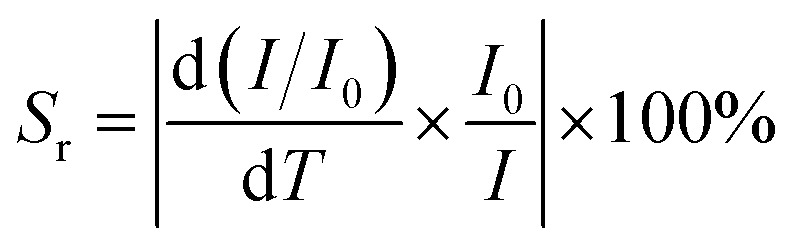
Here, *I* is the PL intensity of the film at a particular temperature and *I*_0_ is the PL intensity of the film at the reference temperature, which is, in this case, the room temperature. The change in *S*_a_ and *S*_r_ values with temperature is shown in Fig. 5c. The sensitivity of the temperature sensor increases as the temperature decreases. The maximum absolute sensitivity was calculated to be 0.032 K^−1^ at 303 K, while the maximum relative sensitivity is 12.5% K^−1^ at 363 K.

We also calculated the response and recovery time, which is defined as the time taken by the sensor to reach 90% of the final equilibrium value, for the FP(B/I)@S@P NC film at both RT and at 70 °C as shown in Fig. S12 (in the ESI†). For the temperature response analysis, the FP(B/I)@S@P film was first kept at RT, and the corresponding PL response was recorded with respect to time. Subsequently, the PL response was recorded when the film was subjected to 70 °C. The response time for the temperature sensor from RT to reach 70 °C was found to be approximately 15 s. Similarly, the recovery time of the sensor to reach from 70 °C to RT is also about 15 s, indicating consistent thermal response kinetics across this temperature range. To have a better understanding of the effect of coating on the sensing performance of the NC film, we also investigated the temperature sensing performance of the FP(B/I)@O NC film to have a comparative analysis before and after double-coating. The corresponding sensitivity plot with temperature is provided in the ESI (see Fig. S13a–c†). No significant change in the sensing performance was observed in the uncoated and double-coated samples. This may be because the sensing mechanism is predominantly governed by the bulk photo-physical properties rather than surface-level interactions. A support table containing a comparative study of luminescent thermometers for room temperature sensing applications is given in Table S3 in ESI.† A photographic image of the fluorescence temperature sensing set-up is shown in Fig. S14 in the ESI† section.

**Fig. 6:**
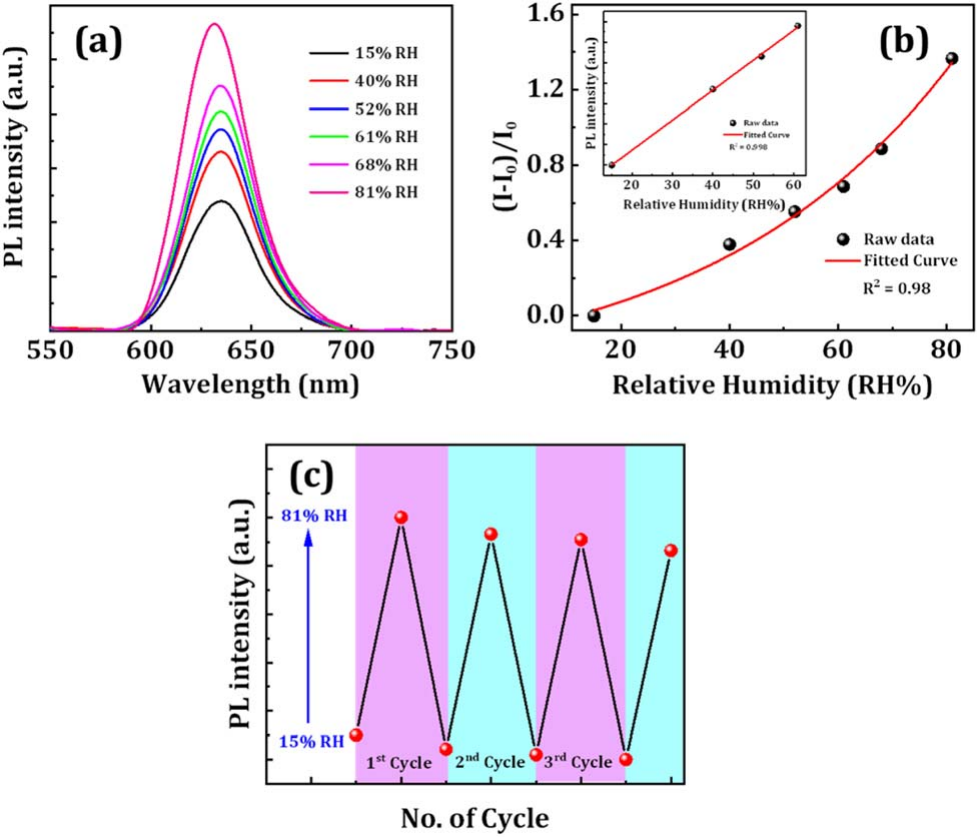
(a) Variation in PL intensity and (b) corresponding fitted curve of the FP(B/I)@S@P NC film under different humidity conditions as shown in legends. (c) Cycle representing retention of PL intensity of the FP(B/I)S@P NC film under high (81%) and low (15%) relative humidity conditions. The inset of figure (b) presents the linear fit of the four lowest humidity conditions where the PL intensity started to change.

We also examined the spectral dependency of FP(B/I)@S@P NC film's emission upon exposure to controlled relative humidity (RH). The experimental set-up for the fluorescent humidity sensor is shown in the ESI (see Fig. S15†). The humidity-dependent fluorescence spectra of films were noted by varying RH from 15% to 81% (see Fig. 6a). The PL intensity of the perovskite film increases with an increase in RH up to ∼80%. In the double-coated FP(B/I)@S@P NC sample, the inherently porous nature of silica and the weak H-bonding between silica and PMMA permit partial moisture ingress into the sample, allowing water molecules to reach the core to interact.^49–51^ At moderate humidity levels, moisture temporarily aids in defect passivation in the FP(B/I)@S@P NC film under UV illumination, thus increasing the carrier recombination rate by the radiative recombination pathway to enhance the PL intensity prior to the onset of degradation under prolonged exposure to elevated humidity.^52,53^Fig. 6b presents the humidity-dependent optical response for the FP(B/I)@S@P NC film. The response is defined using the relation *R* = (*E* − *E*_0_)/*E*_0_, where *E* is the PL intensity of the NC film at different RHs and *E*_0_ is the PL intensity of the film under dry conditions.^54^ The LOD for the humidity sensor was calculated from the calibration curve by taking the lowest four humidity values where the change in the PL intensity occurred (see the inset of Fig. 6b). The sensor's detection limit was calculated using the formula 3*σ*/*S*, where *σ* is the standard deviation and *S* is the slope of the calibration curve. The LOD of the sensor was calculated at ∼5% RH. The FP(B/I)@S@P NC sensor also demonstrated the PL retention capability after exposure to several cycles of dry and wet conditions. We experimented with four such cycles, and the retention of PL intensity for the FP(B/I)@S@P NC film was ∼86% at the end of the 3^rd^ cycle, as presented in Fig. 6c.

We also executed the dynamic response test for FP(B/I)@S@P NC film-based humidity sensors to provide more information about the response time of the fluorescent sensor. To find the response time, the NC film was sequentially kept under dry (low) and relatively high humidity conditions, and the corresponding spectral response with time was recorded. It has been found that the response time of the sensor to reach from low to high humidity conditions is about 5 s, as depicted in Fig. S16 (in the ESI†). For a comparative analysis of the effect of coating on the sensing performance of NC films, we recorded the spectral response of the FP(B/I)@O NC film to various humidity conditions. The corresponding PL variation graph with LOD calculation is shown in Fig. S17.† The LOD value for the FP(B/I)@O NC film was found to be ∼6.7% RH. A support table containing the comparative study of different fluorescent humidity sensors is given in Table S4 in the ESI.†

**Fig. 7:**
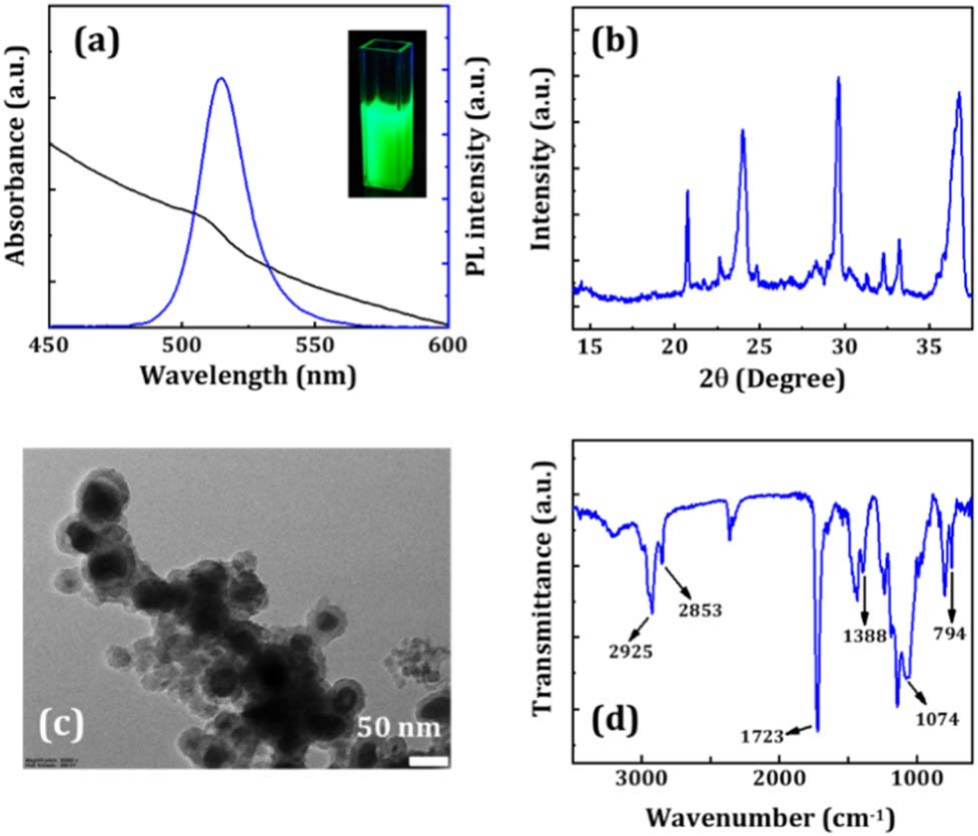
(a) UV-Vis absorption and PL spectra, (b) XRD pattern, (c) TEM image, and (d) FTIR spectrum of FA-Cs10@S@P NCs. The inset of figure (a) presents a photographic image of the NC solution under a UV lamp.

For white light emission, using perovskite NCs with a good color rendering index requires broad coverage of the visible spectrum, which contains green, red, and blue emissions. Following the reported method, we synthesized 10% Cs-doped FAPbBr_3_ NCs as a green emission source encapsulated with silica and PMMA shells (*i.e.*, FA-Cs10@S@P NCs).^36^Fig. 7a presents the UV-Vis absorption and PL spectra of FA-Cs10@S@P NCs. The band edge for the FA-Cs10@S@P NCs is located at around 523 nm, corresponding to a bandgap of ∼2.3 eV. The emission peak for the NCs is located at ∼515 nm (FWHM ∼ 22 nm) with a PLQY of ∼60%. The XRD pattern of the NC film (see Fig. 7b) exhibits peaks at 20.75°, 29.65°, 33.21°, and 36.74° that appear from the reflection from the (011), (002), (021), and (211) lattice planes of the cubic FAPbBr_3_ phase with the *Pm*3̄*m* space group.^36^ Minor XRD peaks at 24.05° and 30.31° are attributed to the diffraction from the monoclinic CsPbBr_3_ phase (PDF # 00-18-0364). Fig. 7c demonstrates the morphology of FA-Cs10@S@P NCs, which shows a core@shell structure in which blurred silica shells can be visible around the dark perovskite structures. The FTIR spectrum of the NC film (see Fig. 7d) reveals two peaks at around 749 and 1074 cm^−1^ that correspond to symmetric stretching of the Si–O bonds and antisymmetric stretching of the Si–O–Si bonds, respectively. The additional 1388 and 1723 cm^−1^ peaks are ascribed to –CO and C=O stretching vibrations of OA, OAm, and the PMMA polymer.^36^

**Fig. 8:**
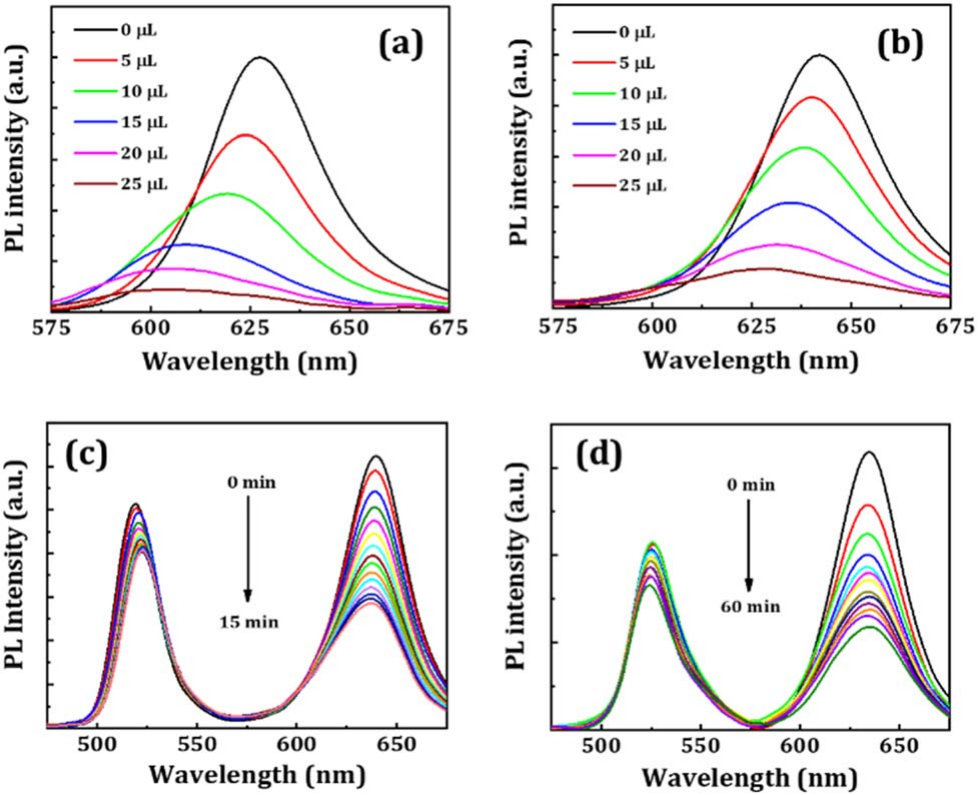
PL spectra of (a) FP(B/I)@S and (b) FP(B/I)@S@P NC solutions after the addition of different volume of TBA-Cl precursor solution, as shown in legends. Change of PL spectra of mixed FA-Cs10@S@P and FP(B/I)@S@P NCs over time in (c) solution phase and (d) film form.

Stability against halide migration is important for practical device-related applications. The stability of the red-emitting NCs was tested against halide migration in the presence of TBA-Cl precursor solution. Here, equal concentrations of NC dispersion (50 μL) were taken into 1.5 mL of toluene, and then equal amounts of TBA-Cl precursor solutions were added in separate glass vials.^36^ The PL intensity of FP(B/I)@O NCs quenched drastically after adding 5 μL TBA-Cl solution, which may be due to the fast degradation of the NCs. Therefore, we continued the experiment with FP(B/I)@S and FP(B/I)@S@P NCs. The changes in the PL spectra of the NCs were recorded as presented in Fig. 8a and b. The PL peak shift and change in emission intensity of the NCs are shown in Fig. S18a and b (ESI†). The emission spectra of these NCs started to shift towards lower wavelength regions with an increase in TBA-Cl solution, and emission intensity was also quenched. The emission peak of the FP(B/I)@S and FP(B/I)@S@P NCs shifted ∼25 and ∼13 nm, respectively, with the incorporation of 25 μL TBA-Cl solution, while the retention of emission intensity was ∼8% and ∼15% respectively. This signifies that coatings inhibit the faster migration of halide ions inside the NCs. Next, we mixed double-coated green-emitting FA-Cs10@S@P and red-emitting FP(B/I)@S@P NC solutions in a quartz cuvette, and the variation in PL spectra was noted for 15 min (see Fig. 8c). The emission spectra of both NCs do not shift significantly, but the PL intensity decreased by ∼21% for FA-Cs10@S@P NCs and ∼54% for FP(B/I)@S@P NCs. Later, we drop-cast the fresh mixed FA-Cs10@S@P and FP(B/I)@S@P NC solutions on a glass substrate and observed the variation of emission spectra for 60 min (see Fig. 8d). The PL intensity of the mixed film started to decrease over time, but minor shifts in peak positions can be noted. We also executed the heat stability test of the film, and the spectra are portrayed in Fig. S19 (ESI†).

**Fig. 9:**
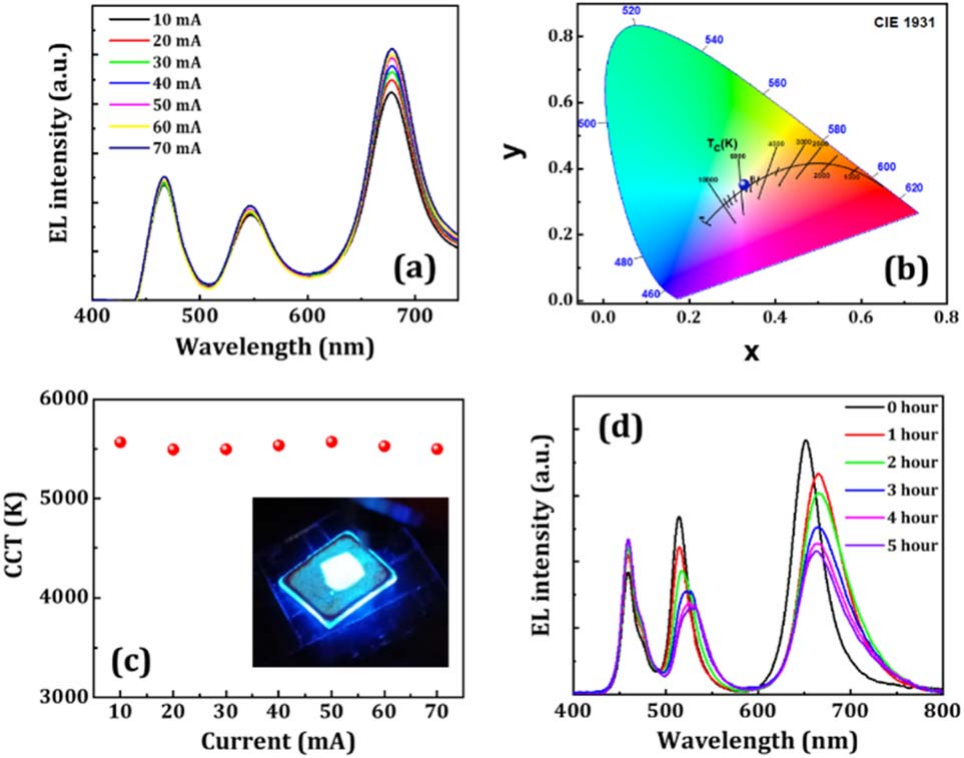
(a) Variation of EL spectra of the WLED device at different driving currents as represented in the legend. (b) Color chromaticity coordinates of the WLED (blue point) at 10 mA driving current. (c) Dependency of the CCT value of the WLED operated at different driving currents. Inset: a photographic image of a working WLED at an external driving current of 10 mA. (d) Change in EL spectra of the WLED device at different operational times as shown in the legend.

Finally, we fabricated down-converted WLED devices by combining the FA-Cs10@S@P and FP(B/I)@S@P NCs. We took FA-Cs10@S@P and FP(B/I)@S@P NCs in a 2 : 1 weight ratio and then spin-coated them on a glass substrate. The WLEDs were fabricated by placing the NC film on a blue LED chip (emission at ∼465 nm) operated through an external bias.^33,39^ The device's electroluminescence (EL) spectra at different driving currents are shown in Fig. 9a. We varied the driving current from 10–70 mA, in which the EL intensity increased with the current. The resulting light emitted from the device is white in color, covering the entire color gamut in the visible spectrum. The CIE color coordinates of the WLED were measured to be (0.33, 0.35) while biased at 10 mA driving current (see Fig. 9b), which are close to those of the ideal white light source. The CRI and CCT values were calculated to be ∼76.85 and ∼5568 K, respectively. The CRI values didn't change over different driving currents. However, the CCT values slightly changed from 5568 to 5500 K at high current, as shown in Fig. 9c. We further verified the operational stability of the WLED device for 5 hours, and the variation in EL spectra is illustrated in Fig. 9d. It has been found that the EL intensity of the WLED decreases with prolonged operation times due to material degradation from excessive heating. The device retained over 50% of the initial PL intensity after 5 hours of continuous operation.

## 4. Conclusions

We synthesized different red-emitting FAPb(Br/I)_3_ NCs *via* the room-temperature LARP synthesis method and encapsulated them with different ligands or shells. The XRD pattern of OA and OAm capped FP(B/I)@O NCs resembles that of the cubic FAPbI_3_ phase, which degrades quickly in the presence of external stimuli. To improve the stability of the NCs, we coated them with different shell materials such as silica and the PMMA polymer. The double-coated FP(B/I)@S@P NCs exhibit red emission having the PL peak position at 642 nm (FWHM ∼ 36 nm). These NCs demonstrated high sensitivity toward temperature and humidity, and were tested as sensors. The temperature sensitivity of the sensor was quantified with an absolute sensitivity (*S*_a_) of 0.032 K^−1^ and relative sensitivity (*S*_r_) of 12.5% K^−1^, over a temperature range of 303 to 363 K. The optical humidity sensor exhibited a detection limit of ∼5% RH. We also synthesized green-emitting double-coated Cs-doped FAPbBr_3_ (*i.e.*, FA-Cs10@S@P) NCs *via* the same LARP method, showing good emission intensity and crystallinity. Finally, we fabricated down-converted WLEDs using hybrid films of double-coated FA-Cs10@S@P and FP(B/I)@S@P NCs. The WLED features CIE color coordinates of (0.33, 0.35) and a correlated color temperature of about 5568 K at 10 mA driving current and displays good operational stability. These findings suggest that FA-based hybrid perovskite NCs have the potential for advanced applications in both sensing and lighting technologies.

## Author contributions

S. B. and K. S. conceived the research idea and planned the experiments. Latika assisted with some material characterization processes. S. B. and K. S. wrote the full manuscript. All authors have given approval to the final version. S. B. led the project.

## Conflicts of interest

There are no conflicts to declare.

## Supplementary Material

NA-OLF-D5NA00412H-s001

## Data Availability

The data supporting this article have been included as part of the ESI.†
