# Supplementary material for: Double-encapsulated red-emitting formamidinium lead halide perovskite nanocrystals for fluorescence sensing and lighting applications
Source: Nanoscale Adv. 2025 Jul 14;7(17):5411–20. doi: 10.1039/d5na00412h (PMC12311843; doi:10.1039/d5na00412h)
Supplement: NA-007-D5NA00412H-s001 [file NA-007-D5NA00412H-s001.pdf]

Supplementary Information (SI) for Nanoscale Advances.
This journal is © The Royal Society of Chemistry 2025

Supporting Information

Double-encapsulated red-emitting formamidinium lead halide perovskite nanocrystals for fluorescent sensing and lighting applications

Kajol Sahoo,[1] Latika,[2] Ramakanta Naik,[1] Saikat Bhaumik [2,*]

1. Synthesis of silica and PMMA encapsulated *Cs*-doped $FAPbBr_3$ NCs: We dissolved 0.045 mmol FABr, 0.005 mmol CsBr, 0.05 mmol $PbBr_2$ in 500 µL DMF in a glass vial. Then, we mixed 100 µL OA and 20 µL OAm into the precursor solutions to form a final precursor solution. 250 µL of the final precursor was added dropwise to the round bottom flask containing 5 mL $CHCl_3$ and 200 µL TEOS. The reaction continued for 6 hours. The NCs mixture was purified in the same way as described for FP(B/I)@O NCs. For post-treatment polymer coating around the silica-coated sample, 50 mg PMMA powder was first mixed in 1 mL toluene and heated at 80 °C under stirring conditions. Then, 2 mL of the synthesized silica-coated sample was added to the prepared PMMA solution and mixed by stirring for 2 hours at RT. The precipitation of the NCs was collected by centrifugation at 6000 rpm for 15 min and dispersed in toluene. The purified NCs were designated FA-Cs10@S@P NCs.

2. Characterization techniques and sample preparations:

2.1. UV-Vis absorption and photoluminescence (PL) measurements: UV-Vis spectrophotometer UV-1900i SHIMADZU was used to record the UV-Vis absorption spectra. Ocean Insight Maya 2000 Pro-high-sensitivity spectrometer was used to record the PL spectra using the excitation wavelength of λ_{ex}= 370 nm for all the samples. These as synthesized FP(B/I)@O, FP(B/I)@S, FP(B/I)@S@P, and FA-Cs10@S@P NCs were diluted in toluene. Further, they were transported to a quartz cuvette for the measurements.

2.2. Powder X-ray diffraction (P-XRD): The concentrated NCs dispersed in toluene were dropped and cast on the well-cleaned glass substrate 1x1 cm. Bruker D8 diffractometer was used to measure the P-XRD with Cu-K_α (λ= 1.54 Å) as incident radiation at 40 kV and 30 mA power. PANalytical Expert's high score plus software was used to analyze the XRD data.

2.3. Transmission electron microscopy (TEM) images: All the samples in toluene with an optimum solution concentration were dropped on the carbon-coated Cu grids with 200 mesh. Jeol-JEM-2100 PLUS microscope was used to measure the TEM operated at 200 kV.

2.4. Fourier transform infrared spectroscopy (FTIR) measurement: FTIR spectra were recorded in thin film mode using a FT/IR-4600 type A spectrometer.

2.5. Stability tests: heat stability, heat-cool, ion migration, and UV stability: The stability tests of all the NCs were carried out using Ocean Insight Maya 2000 Pro high-sensitivity spectrometer with a 370 nm UV excitation source. All the stability tests were done in open-air atmospheric conditions with 55- 65% humidity.

2.6. *X*-ray Photoelectron Spectroscopic measurement (XPS): The XPS analysis was conducted using XPS Analytical Facility (XPS Lab), Department of Chemical Engineering, ICT-Mumbai. The NC thin film was prepared and exposed to a beam of X-rays, which excites the atoms on the sample's surface.

2.7. TRPL measurement: A Pico Second Time Resolved Fluorimeter was used to measure the time-resolved photoluminescence (TRPL) with an excitation source with λ_{ex}= 370 nm. The NCs solutions having the same concentration were placed in a quartz cuvette. The nonlinear least-squares iteration procedure will analyze the fluorescence decay curve profiles using the decay analysis software

2.8. Preparation of TBA-Cl precursor solution: The TBA-Cl precursor solution was prepared by dissolving 5.5 mg TBA-Cl in 5.1 mL toluene, followed by sonication until complete dissolution of the solute. The 5 µL of the solution was added sequentially to the 1.5 mL NCs dispersion in toluene (concentration ~ 2 mg/mL) to carry out further stability tests.

2.9. Preparation of different humidity conditions: Different saturated salt solutions provide different RH values. Here, 5 mL saturated $CaCl_2$, $CuCl_2$, $MgCl_2$, KCl, $CuSO_4$, and K_2SO_4 were each put into a closed chamber to provide different constant RH values of 15%, 40%, 52%, 61%, 68%, and 81%, respectively, standing for 1 hour at 25 °C.[1] The fluorescence profiles of NCs film were recorded after the perovskite humidity sensor was placed in the chamber with each RH for 1 hour.

2.10. PLQY measurement: The PLQY is determined by comparison method by fluorescence quantum yield of coumarin 153 in methanol using the following equation:

$$QY_S = QY_R \times \frac{I_S}{I_R} \times \frac{A_R}{A_S} \times \left(\frac{R_S}{R_R}\right)^2$$

where, QY_S and QY_R represent the PLQY of the sample and reference (i.e., Coumarin 153 in methanol), respectively. I_S is the integrated area under the PL spectrum of the sample, I_R is the integrated area under the PL spectrum of the reference solution, A_R is the absorbance of the reference solution at the PL excitation wavelength (370 nm), A_S is the absorbance of the sample at the sample wavelength. Rs and R_R are the refractive index of the solvents used for the sample dispersion and reference, respectively.

3. Figures:

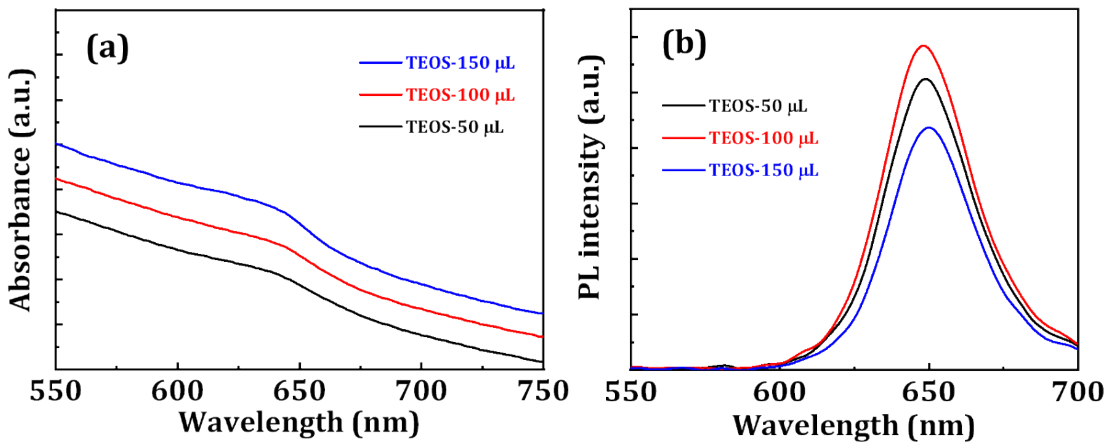

Figure S1: (a) UV-Vis absorption, and **(b)** PL spectra of FP(B/I)@S NCs solutions with different silica content, as shown in legends.

3.1. Fig. S1.

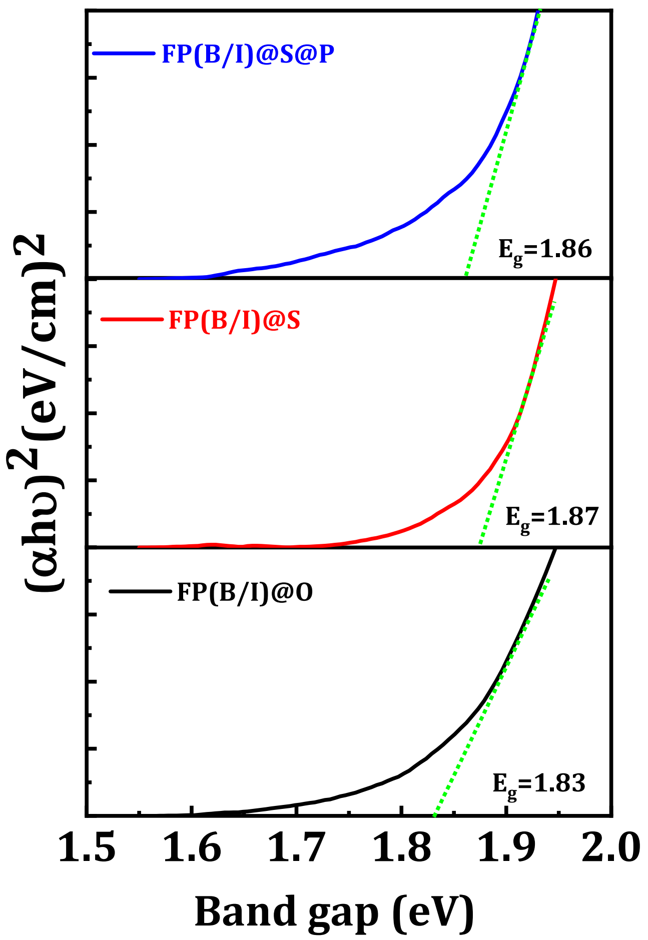

Figure S2: Tauc plot of all NCs as shown in different colors in legend.

3.2. Fig. S2.

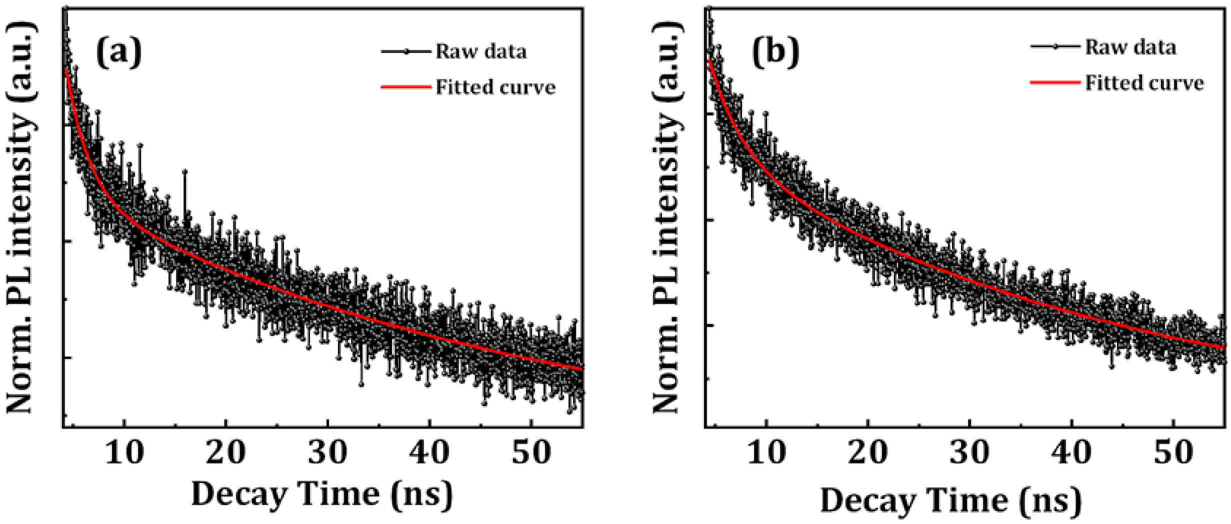

Figure S3: TRPL spectra of **(a)** FP(B/I)@O and **(b)** FP(B/I)@S@P NCs dispersions. The corresponding spectra were fitted with a bi-exponential decay fitting.

3.3. Fig. S3.

Table S1: PL decay lifetime of different NCs.

Sample Name	A_1	τ_1 (ns)	A_2	τ_2 (ns)	τ_{avg} (ns)
FP(B/I)@O	0.11281	2.31731	0.54685	47.41397	46.96
FP(B/I)@S@P	0.19832	3.28029	0.64803	41.38379	40.48

3.4. Fig.S4.

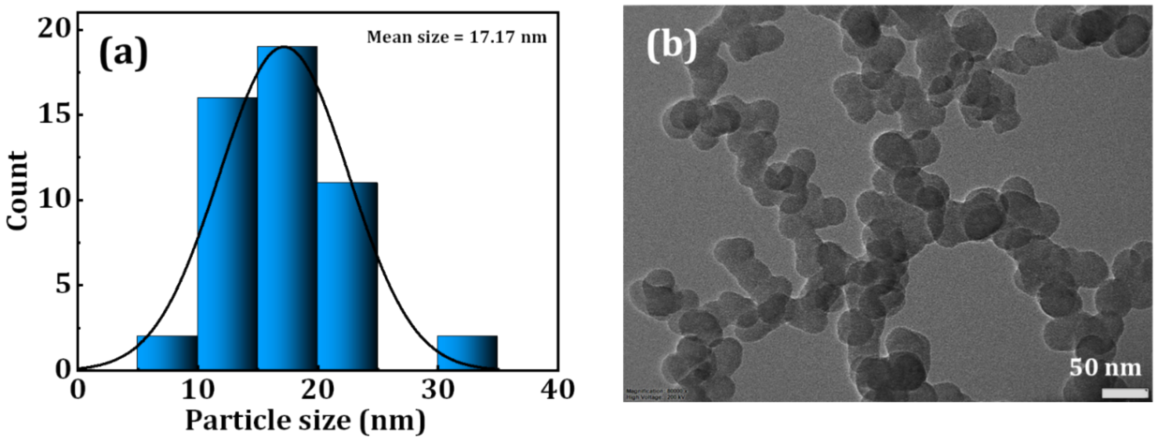

Figure S4: (a) Size distribution of FP(B/I)@O NCs, **(b)** TEM image of FP(B/I)@S@P NCs.

3.5. Fig. S5.

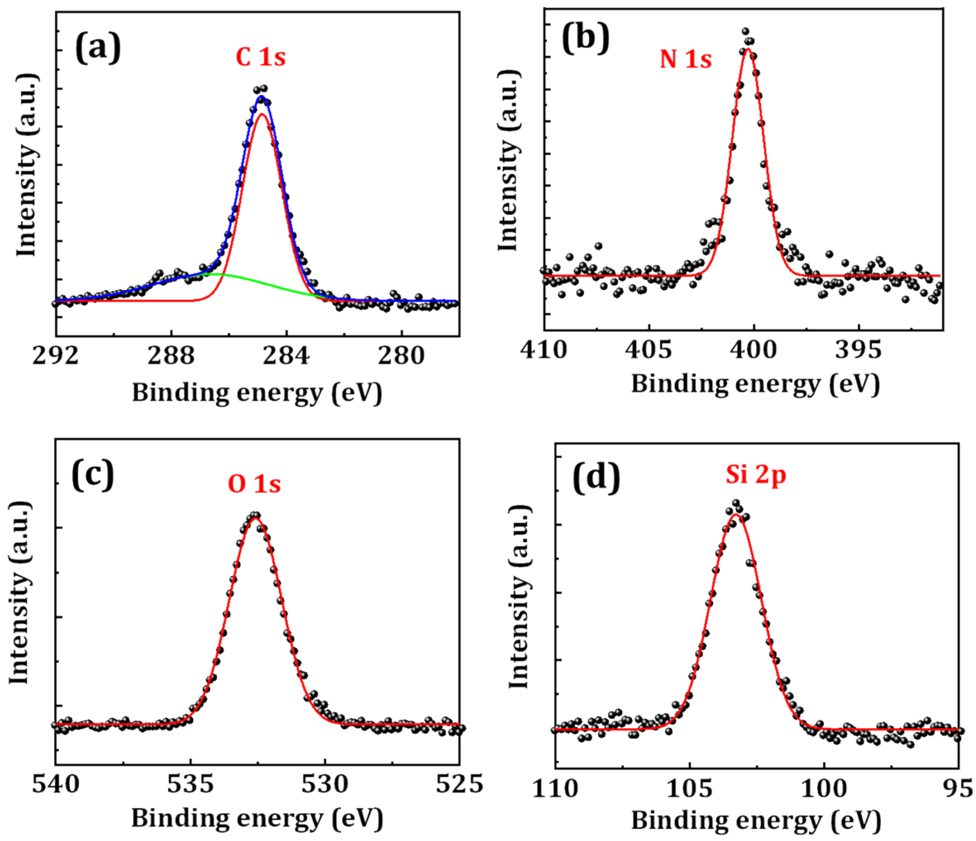

Figure S5: HR-XPS spectra of **(a)** *C* 1s, **(b)** *N* 1s, **(c)** *O* 1s, and **(d)** *Si* 2p chemical states of FP(B/I)@S@P NCs.

Elements	Oxidation state	Peak position (eV)	FWHM (eV)	Relative intensity (a.u.)	Intensity ratio of spin-orbit doublet
Pb	$4f_{7/2}$	138.28	1.40	10696.33	1.25
	$4f_{5/2}$	143.15	1.45	8525.00	
I	$3d_{5/2}$	618.97	1.63	12092.35	1.44
	$3d_{3/2}$	630.45	1.66	8342.43	
C	1s	284.84	1.61	9830.50	-
	1s	286.64	4.76	5147.56	
Br	3d	68.60	1.95	2626.54	-
O	1s	532.58	2.28	12432.81	-
N	1s	400.29	1.76	1558.71	-
Si	2p	103.28	2.22	3161.25	-

Table S2: Fitted parameters of HR-XPS spectra for FP(B/I)@S@P NCs.

3.6. Fig. S6.

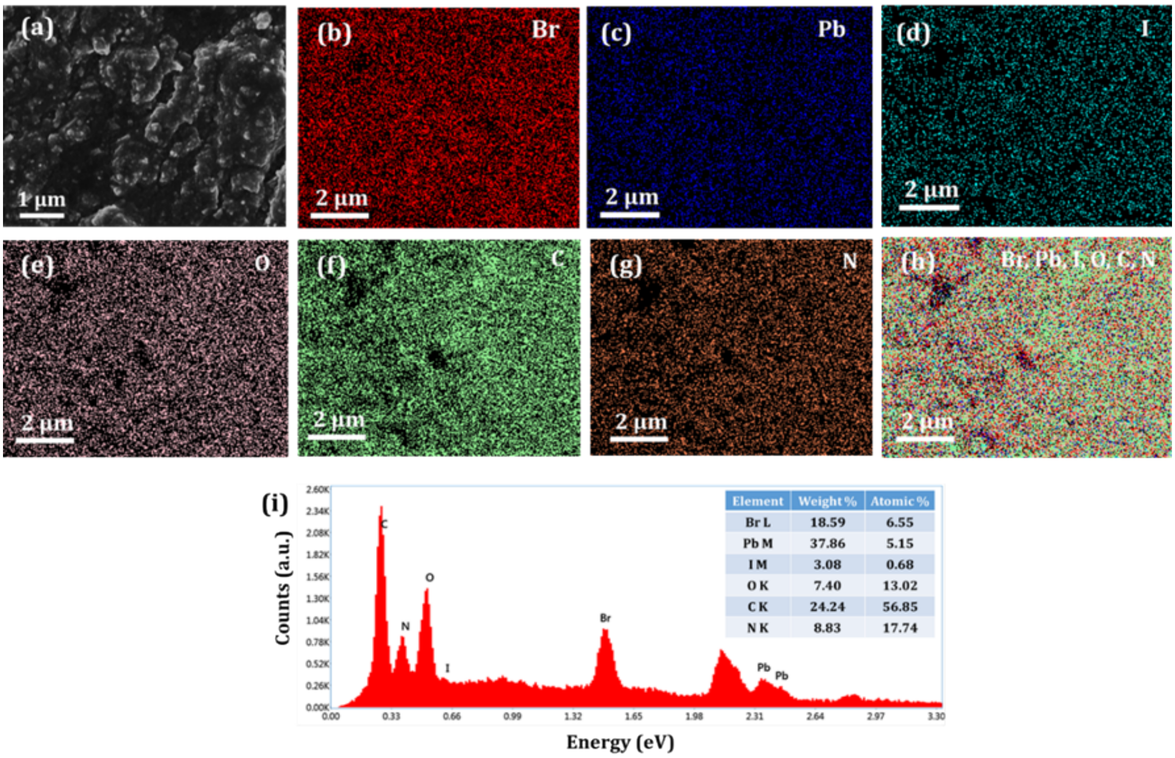

Figure S6: **(a)** FESEM image of FP(B/I)@O NC film on glass substrate, and **(b-h)** corresponding elemental mapping showing the presence of various elements. **(i)** EDS spectrum of FP(B/I)@O NC film showing all the elements present on the surface. Inset: EDS data obtained from FESEM image showing both the weight and atomic percentage of different elements.

3.7. Fig. S7.

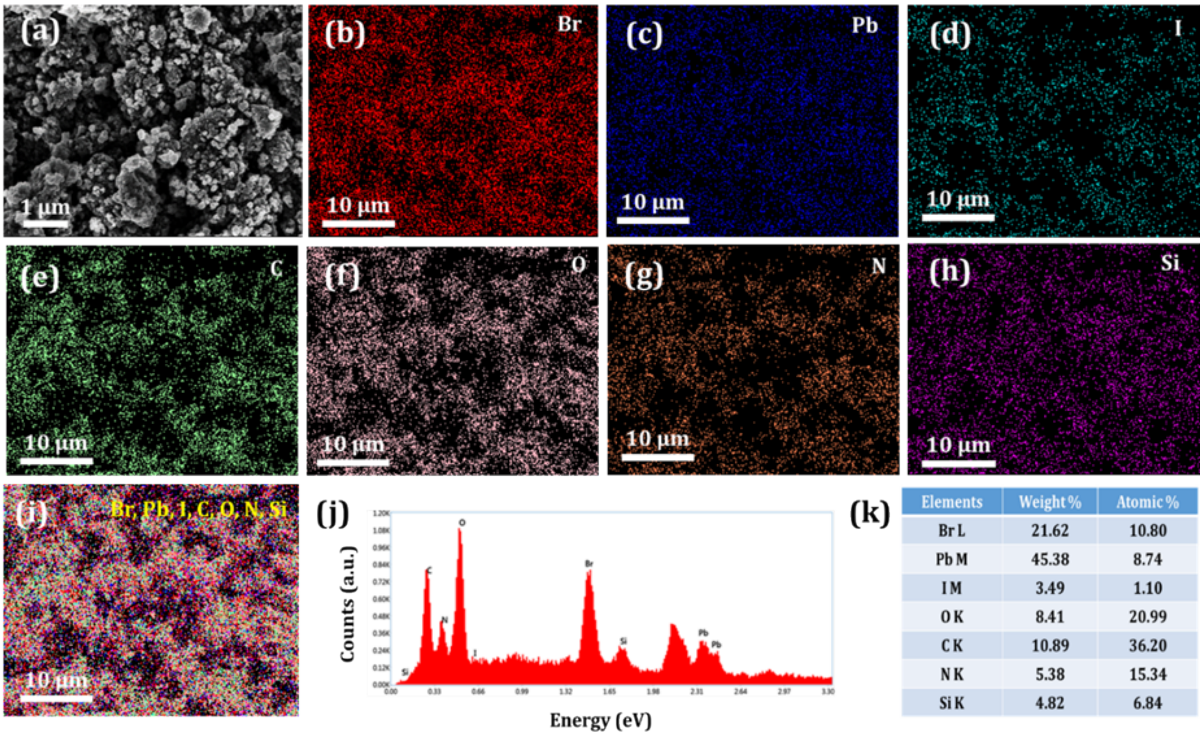

Elements	Weight%	Atomic%
Br L	21.62	10.80
Pb M	45.38	8.74
I M	3.49	1.10
O K	8.41	20.99
C K	10.89	36.20
N K	5.38	15.34
Si K	4.82	6.84

Figure S7: (a) FESEM image of FP(B/I)@S NC film on glass substrate, and **(b-i)** corresponding elemental mapping showing the presence of various elements. **(j)** EDS spectrum of FP(B/I)@S NC film showing all the elements present on the surface. **(k)** EDS data obtained from FESEM image showing both the weight and atomic percentage of different elements.

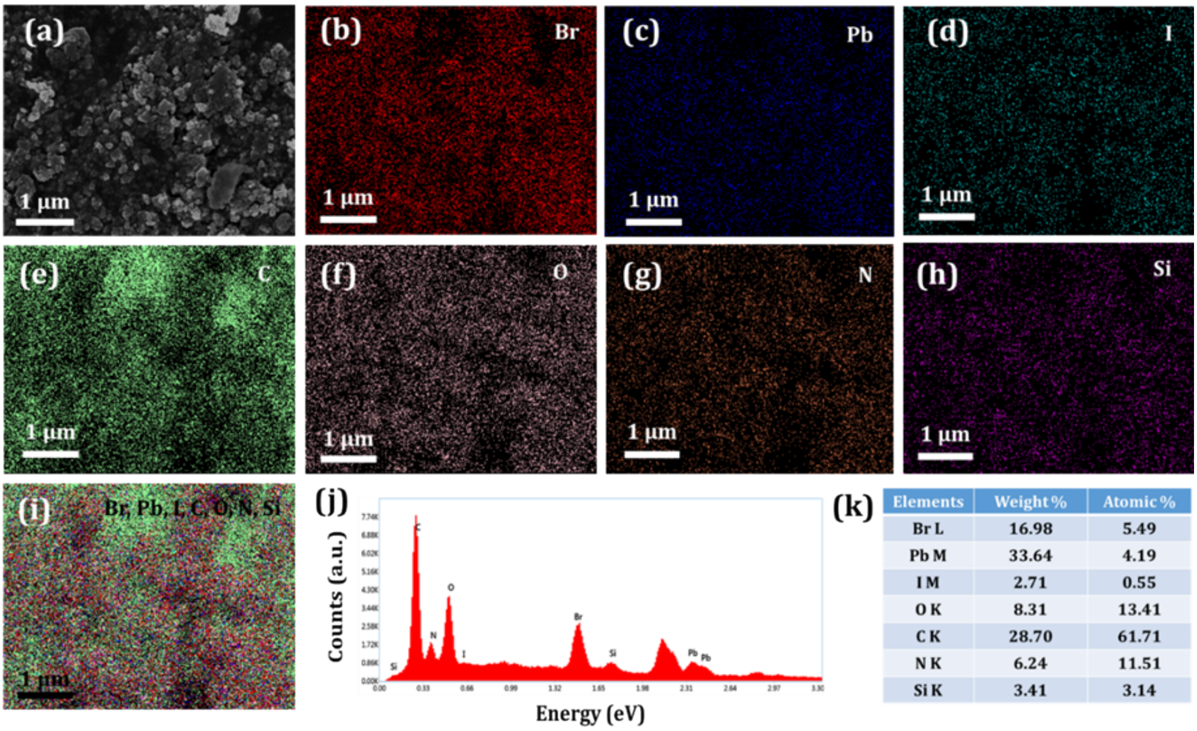

Elements	Weight%	Atomic%
Br L	16.98	5.49
Pb M	33.64	4.19
I M	2.71	0.55
O K	8.31	13.41
C K	28.70	61.71
N K	6.24	11.51
Si K	3.41	3.14

Figure S8: (a) FESEM image of FP(B/I)@S@P NC film on glass substrate, and **(b-i)** corresponding elemental mapping showing the presence of various elements. **(j)** EDS spectrum of FP(B/I)@S@P NC film showing all the elements present on the surface. **(k)** EDS data obtained from FESEM image showing both the weight and atomic percentage of different elements.

3.8. Fig. S8.

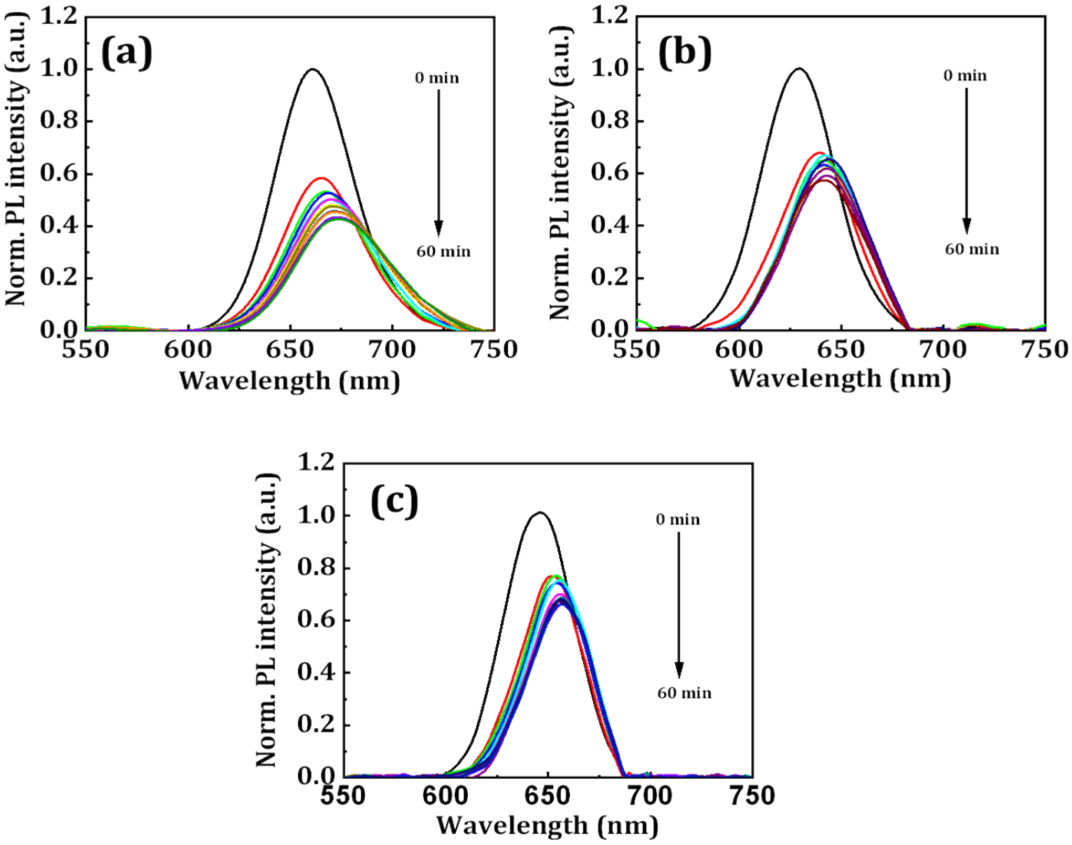

Figure S9: Variation in PL intensity of **(a)** FP(B/I)@O**,** **(b)** FP(B/I)@S, **(c)** FP(B/I)@S@P NC films when exposed to continuous UV irradiation for 1 hour.

3.9. Fig. S9.

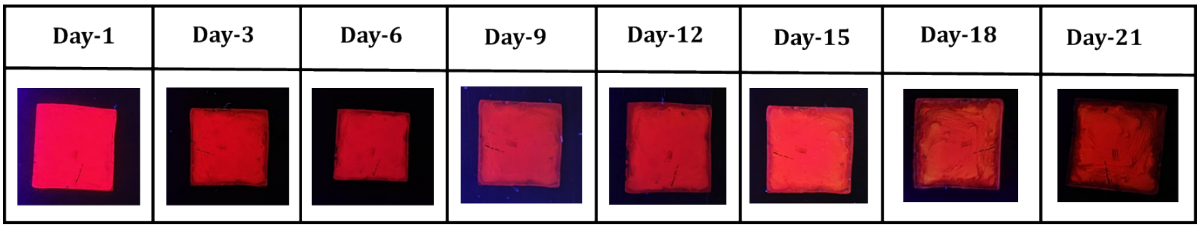

Figure S10: Photographic images of FP(B/I)@S@P NC film under UV-illumination with time.

3.10. Fig. S10.

3.11. Fig.S11.

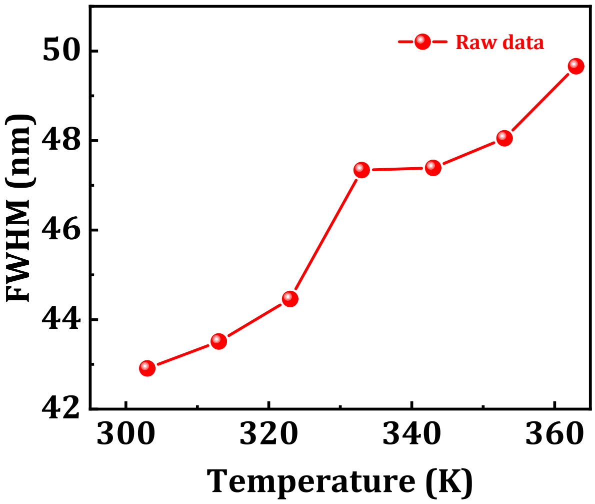

Figure S11: Variation of FWHM of FP(B/I)@S@P NC film with increase in temperature.

3.12. Fig.S12.

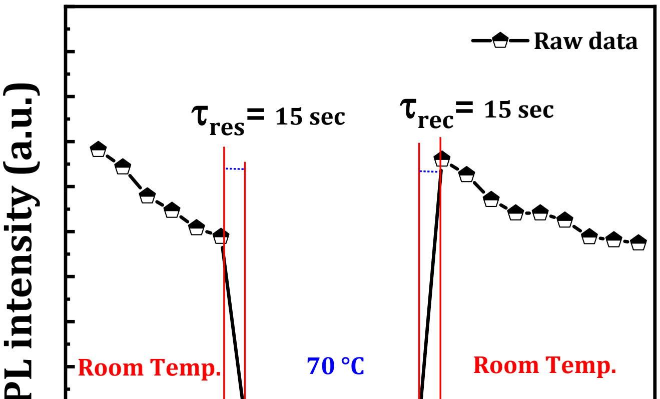

3.13. Fig.S13.

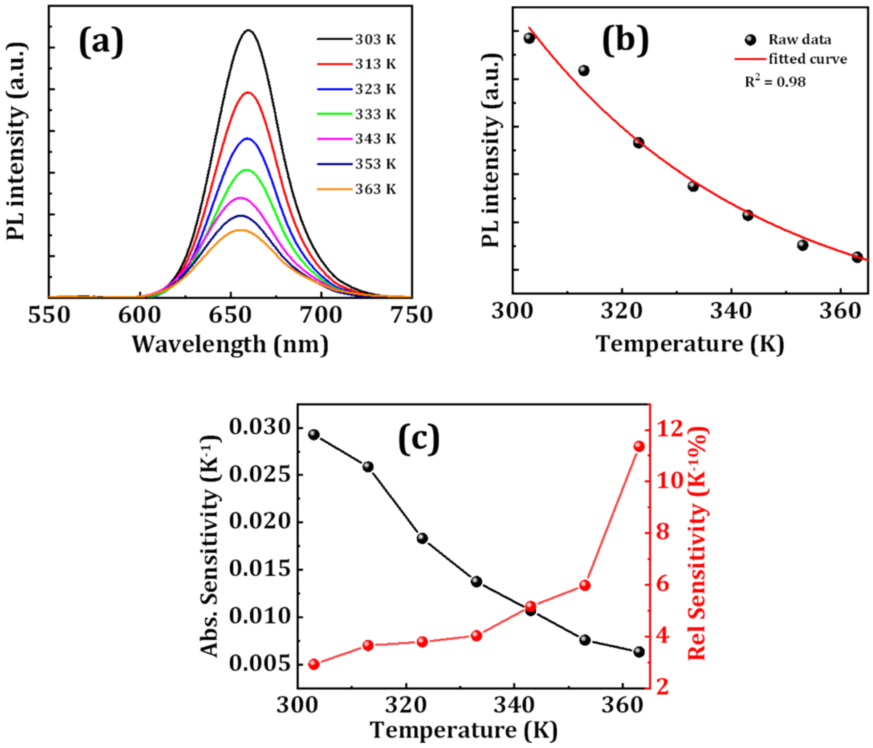

Figure 13: (a) PL spectra of FP(B/I)@O NC film while placed on a hot plate maintained at different temperature, **(b)** fitted data of PL intensity of FP(B/I)@O NC film at different temperature, **(c)** plot of sensitivity of the temperature sensor as a function of temperature.

Table S3: A support table containing a comparative study of luminescent thermometers for room temperature sensing applications with different sensitivity.

Name of fluorescent probe	Linear range	Relative sensitivity	Reference

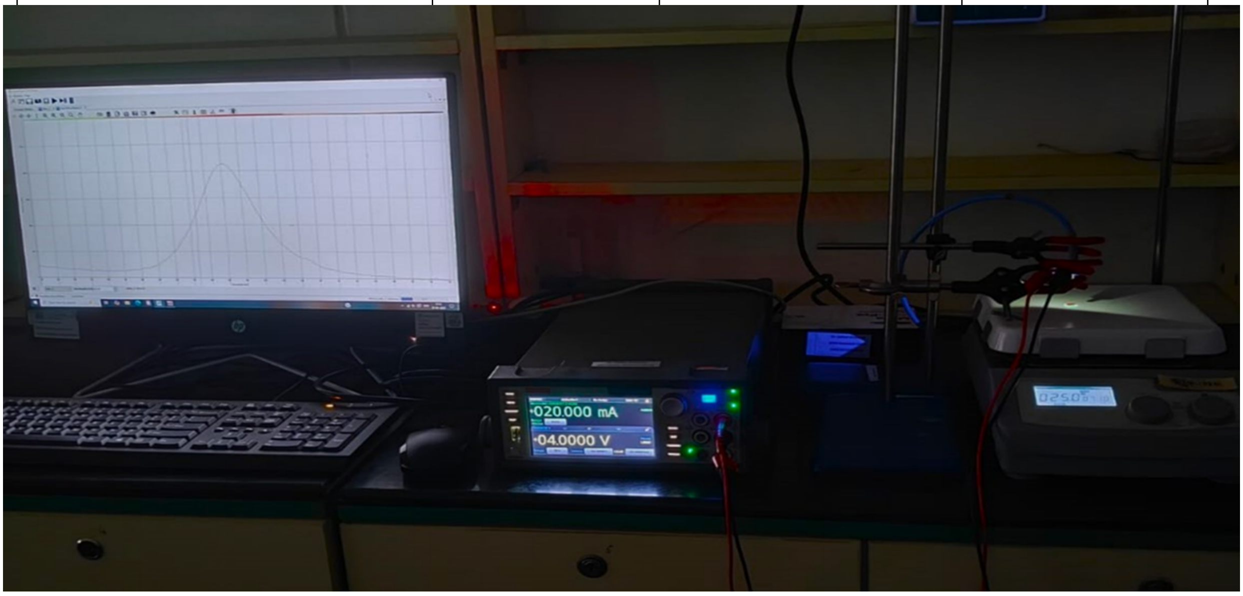

Figure S14: Photographic image of fluorescent temperature sensing setup.

3.14. Fig. S14.

3.15. Fig. S15.

3.16. Fig. S16.

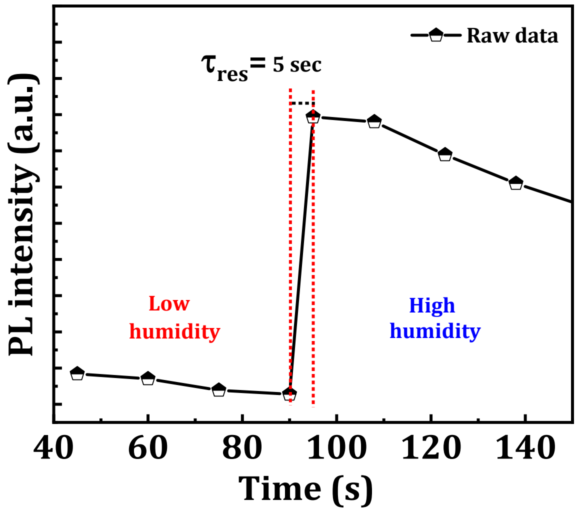

Figure S16: Dynamic response curve of FP(B/I)@S@P humidity sensor.

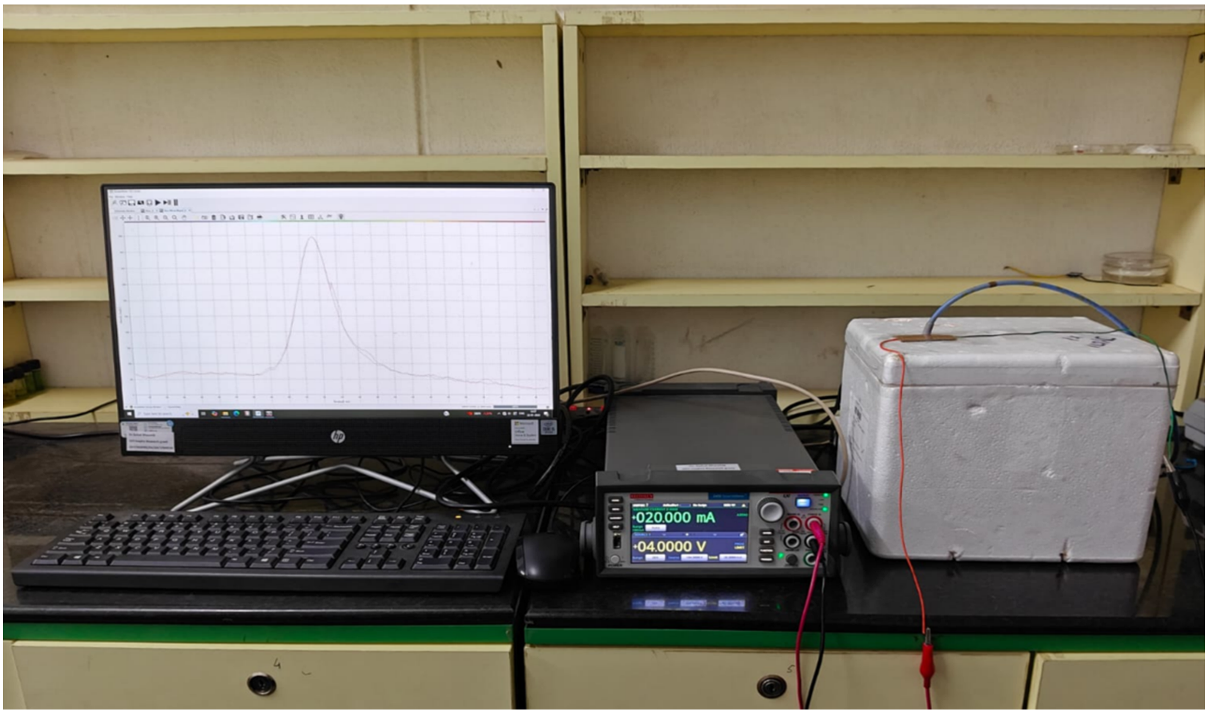

Figure S15: Photographic image of fluorescent humidity sensing setup.

3.17. Fig. S17.

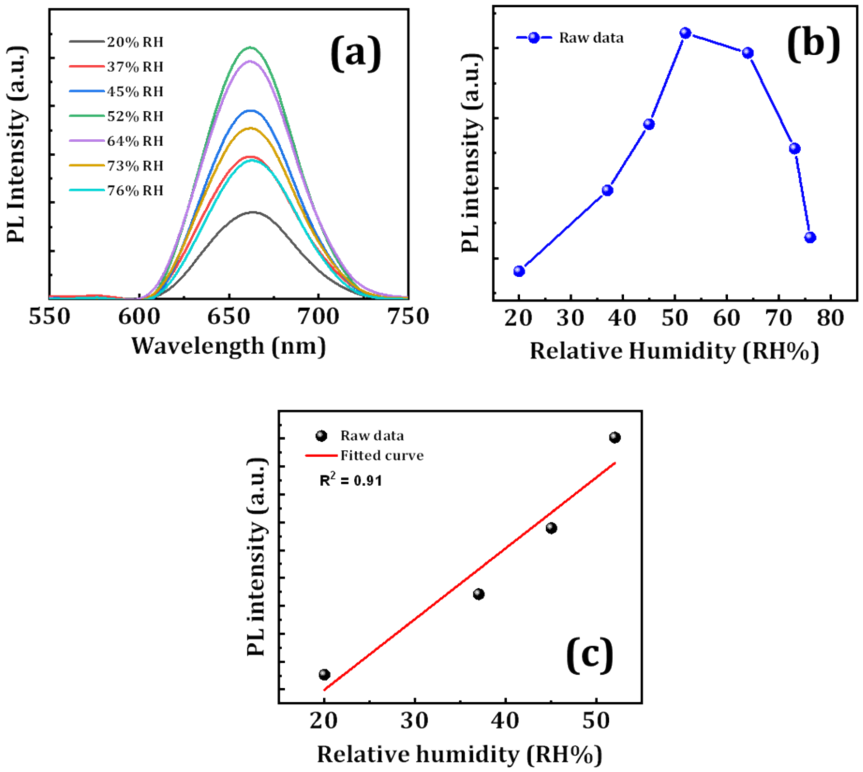

Figure S17: (a) PL spectra of FP(B/I)@O NC film at different humidity conditions, **(b)** corresponding ray diagram, and **(c)** linear fitting of the four lowest humidity conditions where the PL started to vary.

T

with different detection limits.

Name of fluorescent probe	Linear range	Detection limit	Reference
$CsPbBr_3$ NCs	33-98% RH	12% RH	5
Cs_4PbBr_6 Nanoarray	10-86% RH	9.5% RH	6
Poly[1-phenyl-2 (p-trimethylsilyl) phenylacetylene]	0-100% RH	20% RH	7
FP(B/I)@O NCs	21-76% RH	6.7% RH	This work
FP(B/I)@S@P NCs	15-81% RH	5% RH	This work

3.18. Fig. S18.

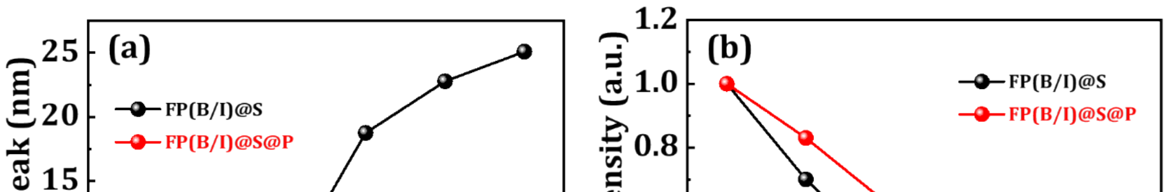

3.19. Fig. S19.

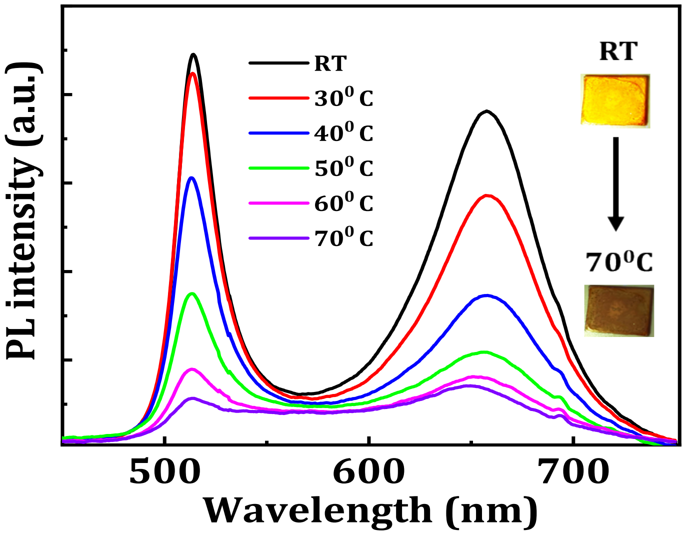

Figure S19: PL spectra of hybrid film grown from FP(B/I)@S@P and FA-Cs10@S@P NCs mixture at different temperatures as represented in legend. Inset: photographic images of the film at room temperature and at 70 ºC.

References:

1. J. F. Young, *J. Appl. Chem.*, 1967, **17**, 241-245.
2. B. Zhuang, Y. Liu, S. Yuan, H. Huang, J. Chen and D. Chen, *Nanoscale*, 2019, **11**, 15010-15016.
3. J. Liu, Y. Zhao, X. Li, J. Wu, Y. Han, X. Zhang and Y. Xu, *Cryst. Growth Des.*, 2020, **20**, 454-459.
4. K. Sahoo, S. Kumar, A. Mohapatra, N. K. Dubey, R. Naik, C. Goswami and S. Bhaumik, *J. Mater. Chem. C*, 2024, **12**, 17315-17327.
5. X. Xiang, H. Ouyang, J. Li and Z. Fu, *Sens. Actuators B Chem.*, 2021, **346**, 130547.
6. Y. Wei, Y. Liu, Y. Zhang, J. Pan, S. Pan, Y. Wei, B. Pan, Z. Lu and X. Xing, *J. Mater. Chem. C*, 2024, **12**, 4054-4061.
7. M. Trigo-López, A. Muñoz, S. Ibeas, F. C. García, F. Serna and J. M. García, *Sens. Actuators B Chem.*, 2014, **191**, 233-238.
